# Sexual and gender-based violence in areas of armed conflict: a systematic review of mental health and psychosocial support interventions

**DOI:** 10.1186/1752-1505-7-16

**Published:** 2013-08-05

**Authors:** Wietse A Tol, Vivi Stavrou, M Claire Greene, Christina Mergenthaler, Mark van Ommeren, Claudia García Moreno

**Affiliations:** 1 Department of Mental Health, Johns Hopkins Bloomberg School of Public Health, 624 N Broadway, Hampton House Room 863, Baltimore, MD 21205-1996, USA; 2 Columbia Group for Children in Adversity, Columbia University, New York, NY, USA; 3 Global Health Initiative, Yale University, New Haven, CT, USA; 4 Department of Mental Health and Substance Abuse, World Health Organization, Geneva, Switzerland; 5 Department of Reproductive Health & Research, World Health Organization, Geneva, Switzerland

**Keywords:** Armed conflict, Sexual violence, Gender-based violence, Mental health, Psychosocial interventions, Prevention, Treatment evaluation, Effectiveness, Efficacy

## Abstract

**Background:**

Sexual and other forms of gender-based violence are common in conflict settings and are known risk factors for mental health and psychosocial wellbeing. We present findings from a systematic review of the academic and grey literature focused on the effectiveness of mental health and psychosocial support interventions for populations exposed to sexual and other forms of gender-based violence in the context of armed conflicts.

**Methods:**

We searched the Cochrane Database of Systematic Reviews, Cochrane Controlled Trials Register, PubMed/ Medline, psycINFO, and PILOTS, as well as grey literature to search for evaluations of interventions, without date limitations.

**Results:**

Out of 5,684 returned records 189 full text papers were assessed for eligibility. Seven studies met inclusion criteria: 1 non-randomized controlled study; 3 non-controlled pre- post-test designs; 1 retrospective cohort with a matched comparison group; and 2 case studies. Studies were conducted in West and Central Africa; Albania; UK and USA, included female participants, and focused on individual and group counseling; combined psychological, medical, social and economic interventions; and cognitive behavioral therapy (two single case studies).

**Conclusions:**

The seven studies, while very limited, tentatively suggest beneficial effects of mental health and psychosocial interventions for this population, and show feasibility of evaluation and implementation of such interventions in real-life settings through partnerships with humanitarian organizations. Robust conclusions on the effectiveness of particular approaches are not possible on the basis of current evidence. More rigorous research is urgently needed.

## Background

Since the end of World War II, 248 armed conflicts have been recorded in 153 locations [1]. Sexual violence has long been part of armed conflicts, with great variation as to scale, type of violence, who is targeted and the extent to which there exists a specific tactic to commit such violence [2]. Sexual violence has been defined in the World Report on Violence and Health as “any sexual act, attempt to obtain a sexual act, unwanted sexual comments or advances, or acts to traffic, or otherwise directed, against a person’s sexuality using coercion, by any person regardless of their relationship to the victim, in any setting, including but not limited to home and work” [3]. Gender-based violence is a broader umbrella term referring to any harmful act that is perpetrated against a person’s will, and that is based on socially ascribed (gender) differences between males and females, which in most settings privilege men. Rates of sexual and other forms of gender-based violence are reported to be higher in areas of armed conflict than in non-conflict affected settings [4], but establishing exact rates is challenging [5]. Comparing rates is even more difficult, given different methodologies and measures used in surveys. Rape, for example, was reported by 11% of displaced women surveyed in Colombia; 19% of surveyed women in Burundi; 25% of women surveyed in Azerbaijan; and 39% of women during the Rwandan genocide [6,7]. Sexual violence in conflict is not restricted to rape, nor does conflict-related sexual violence end when conflicts do. In conflict settings rates of intimate partner violence are often higher than rates of wartime rape and sexual violence perpetrated by individuals outside the home [8].

Sexual and other forms of gender-based violence can have multiple consequences for survivors, including social impacts and negative health outcomes. Recorded social impacts include rejection by family and community, strain on marital relations, impaired parenting skills, children born as a result of rape, exclusion from schools and jobs, being perceived as unfit for marriage, further violence, repeated assault, and isolation [9,10]. Health consequences include sexually transmitted infections and HIV, unwanted pregnancies, abortions, gynecological problems, physical injuries, and maternal mortality [11]. In addition, high prevalence of psychological distress and mental disorders has been documented in survivors of sexual and gender-based violence in areas of armed conflict. Reported mental disorders include anxiety disorders (including Posttraumatic Stress Disorder [PTSD]), major depressive disorder, medically unexplained complaints, substance use disorders and suicidal ideation [12].

International consensus guidelines for prevention and response to sexual and other forms of gender-based violence have been published by the Inter-Agency Standing Committee (IASC) [13]. These guidelines include a section with recommendations relevant to mental health and wellbeing, such as mobilizing community resources; providing emotional support in health and community services; discussing coping strategies; improving family support, and promoting social reintegration [7]. In addition, IASC guidelines have been issued on mental health and psychosocial interventions for emergency settings in general. The composite term mental health and psychosocial support (MHPSS) is defined in these guidelines as “any type of local or outside support that aims to protect or promote psychosocial well-being and/or prevent or treat mental disorder” [13].

Despite the increasing use of MHPSS interventions and publication of consensus guidelines on good practice, a wide gap has been reported between popular practices and knowledge on effectiveness of interventions in this field [14]. The aim of this systematic review was to identify and summarize evaluations of interventions that aimed to prevent mental disorders, promote mental health and wellbeing, or treat psychological distress and mental disorders in adult populations surviving sexual and other forms of gender-based violence in the context of armed conflict. A summary of knowledge on the effectiveness of intervention strategies should assist practitioners in selection of interventions with proven effectiveness and avoiding interventions that have been shown not to be associated with improvements in mental health and wellbeing. It can also help to identify current gaps in knowledge that need to be addressed in future studies.

## Methods

### Search strategy

In line with guidelines based on international consensus [7,13], we applied an inclusive definition of mental health and wellbeing, including psychological symptoms as well as broader aspects of social and emotional functioning. We searched academic peer reviewed literature and grey literature. With regard to the grey literature, we followed a two-step procedure in accordance with a recent systematic review of mental health and psychosocial support in humanitarian settings [14]. First, we identified countries/territories in which armed conflicts were systematically recorded between 2001 and 2009 (the latest year for which data was available at the time of the search. Data was obtained from the International Peace Research Institute (the Uppsala Conflict Data Program) [15-23] and the Heidelberg Institute for International Conflict Research [24-27]. Altogether we identified 53 territories/ countries in which armed conflicts were documented in this period (n = 24 [45.3%] in Africa, n = 12 [22.6%] in Asia, n = 7 [13.2%] in the Middle East, n = 5 [9.4%] in the Americas, and n = 5 [9.4%] in Europe).

Second, we searched the grey literature (i.e. evaluations published on websites, humanitarian reports, etc.) and academic literature. With regard to grey literature, we searched the internet using identified territories in the first steps as keywords, in combination with Boolean phrases including categories of keywords to narrow the search to: (a) *sexual and gender-based violence* (“sexual violence” or “sexual and gender-based violence” or “gender-based violence” or “sexual exploitation” or “sexual abuse” or “rape” or “domestic violence” or “trafficking” or “intimate partner violence”); (b) *mental health and wellbeing* (“mental disorders” or “psychosocial wellbeing” or “emotional wellbeing” or “social wellbeing” or “psychosocial distress” or “PTSD” or “anxiety” or “depression” or “dissociation” or “substance use” or “substance abuse” or “somatoform disorder” or “medically unexplained complaints” or “psychosomatic”; and (c) a broad range of *interventions* (e.g. “intervention” or “response” or “project” or “program” or “services” or “prevention” or “treatment” or “protection” or “social support” or “psychological support” or “assessment” or “monitoring” or “evaluation”). Internet searches using Google were limited to the first 200 hits.

Using the website of the Active Learning Network for Accountability and Performance in Humanitarian Action’s (ALNAP; http://www.alnap.org) advanced search function, we searched the countries / territories described above using a smaller set of keywords after piloting. Finally, we searched 14 websites of key agencies and initiatives in this field (selection based on our searches using Google and ALNAP) for relevant reports. These searches were conducted between May 13 and August 30, 2011.

For the academic literature we followed a slightly different search strategy, to account for the different terminology used by researchers and practitioners. First, we did not use countries/ territories as keywords, but rather used the terms “armed conflict”, “political violence” and “war”. Second, we added the terms “efficacy” and “effectiveness”, as we expected these would be more commonly employed by researchers publishing in the academic literature. We searched the following databases between June 17 and July 10, 2011: Cochrane Database of Systematic Reviews, Cochrane Controlled Trials Register, PubMed/ Medline, psycINFO, and PILOTS.

In addition, we searched the reference lists of a number of relevant reviews [28-33] and the reference lists of included evaluation studies. We contacted key authors in the field, and all authors of included studies, to find out whether they were aware of further studies that would meet inclusion criteria.

### Inclusion and exclusion criteria

We included intervention evaluations if they described the evaluation of prevention or treatment of mental disorders and/or the promotion and protection of psychosocial wellbeing with populations exposed to sexual violence in an armed conflict setting. We included studies if they: (1) specifically noted the context of armed conflict; (2) assessed sexual and gender-based violence in accordance with above cited definitions; (3) described procedures which were followed to prevent mental disorders, promote mental health and wellbeing, or treat psychological distress or mental disorders (for psychosocial support interventions, evaluation studies had to include at least one quantifiable measure of three core psychosocial well-being domains: emotional and social wellbeing; acquisition of skills and knowledge) [34]; and (4) reported evaluation methodology, including either qualitative process measures (e.g. client perceptions of intervention and intervention changes, clinician attitudes, adherence), quantitative outcome measures (e.g. pre- and posttests on screening questionnaires and diagnostic interviews) or a combination of these. We excluded short descriptions of interventions in quarterly or annual reports; opinion pieces; letters to editors; book reviews; manuals; toolkits; and program descriptions without evaluations. We searched without date limitations, but limited our search to reports in English. Because we anticipated that there may be few highly rigorous evaluation studies in this area and to provide a detailed picture of conducted evaluations, we opted for an inclusive strategy and did not exclude evaluation studies on the basis of study quality.

### Data analysis

**Figure 1:**
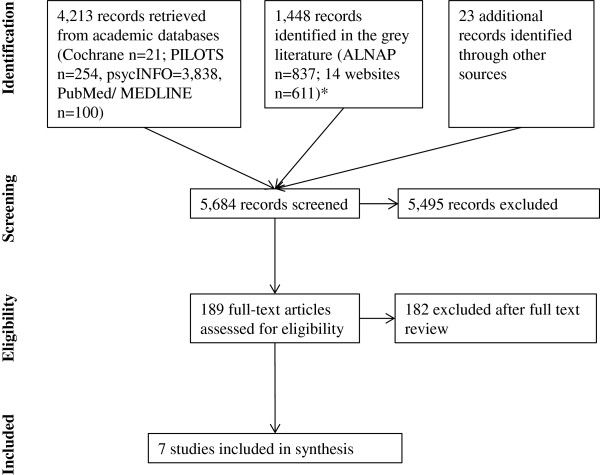
Systematic review flow chart.

After (a) excluding all articles returned by the searches that were clearly not relevant based on abstract and title and (b) excluding duplicate records, two reviewers independently reviewed all manuscripts in full for inclusion and exclusion criteria (for a flowchart see Figure 1). If two reviewers disagreed on relevance of a manuscript, a third reviewer was consulted.

Information reported in included articles was subsequently summarized in an Excel spreadsheet that listed: (a) study type (qualitative, quantitative, or mixed methods); (b) evaluation objectives and hypotheses; (c) research site; (d) study design; (e) selection (random sample, help-seeking, convenience sample, snowball sample, etc.); (f) sample characteristics (age, sex, type of violence); (g) sample size; (h) outcomes of interest and measures used; (i) analytic approach; (j) major results (including any quantitative changes observed); and (k) recommendations.

Quality of papers was assessed using the Downs & Black’s checklist for the assessment of the methodological quality both of randomized and non-randomized studies of health care interventions, which systematically assesses quality of reporting on issues ranging from research aims, power calculations, intervention, and outcome instruments [35]. The checklist was judged to not apply to the two n = 1 studies. Two raters independently scored the 7 included studies using the checklist and compared their scores. Any differences were resolved through discussion. The final score in Table 1 represents the amount of quality items on which a positive score was agreed given the total amount of applicable items (details of scoring available upon request).

**Table 1:** Characteristics of included studies

**Authors, year**	**Setting**	**Design**	**Study conditions**	**Participants (at baseline)**	**Outcomes assessed**	**Quality assessment**
Lekskes, Van Hooren & De Beus [36]	Liberia	Non-randomized controlled trial (mixed methods)	(1) individual and group counseling; (2) support groups and skills training; (3) waitlisted control	N = 66; 100% female; 68% survived sexual violence; average 34 years	Harvard Trauma Questionnaire; observation, semi-structured interviews, focus group discussions	12 out of 27
Bolton [37]	DRC	Pre-, posttest, no control (mixed methods)	Diverse psychosocial and economic interventions	N = 240; 100% female; survived sexual and gender-based violence; average 35 years	Locally developed questionnaire, including questions on functioning and psychological difficulties (fear and anxiety; ill treatment, shame and stigma; depressive symptoms)	15 out of 27
Hustache et al. [38]	Republic of Congo	Pre-, posttest, no control (quantitative)	Medical care and psychological support	N = 178; help-seeking women > 15 years, raped by unknown person in military clothes	Global Assessment of Functioning (pre- and post-test, n = 56); DSM-IV diagnosis (pre-test only, n = 159); Trauma Screening Questionnaire (post-test only, n = 64); locally developed psychological symptom checklist (post-test only, n = 64)	14 out of 27
Plester, [39]	Albania	Pre-, posttest, no control (quantitative)	Group counseling, individual sessions where necessary	N = 39; 100% female; 1 group politically persecuted, 1 group from slum areas, 1 group female-headed households; average 43 years	Screen for Posttraumatic Stress Symptoms; Brief Symptom Inventory; locally developed empowerment questionnaire	12 out of 27
Ager et al. [40]	Sierra Leone	Retrospective cohort with matched comparison group (mixed methods)	Psychosocial interventions, including traditional healing, medical services, skills-training, micro-credit loans, and community awareness raising	N = 142; 100% female; former combatants; age range 17 – 25 years	Locally developed structured interviews focused on six community integration indicators	16 out of 27
Vickers [41]	UK	Single case study	Cognitive behavioral therapy	Female refugee from Africa (country NR); rape survivor; 14 years	Post-Traumatic Diagnosis Scale (pre-test, session 6, 8, 10, 15, 16)	NA
Schulz, Marovic-Johnson & Huber [42]	USA	Single case study	Cognitive behavioral therapy (cognitive processing therapy); anti-depressant	Female Bosnian refugee; repeated sexual and physical violence and rape survivor; 64 years	Pre-, during (two months), and end of treatment assessment using clinical diagnosis; PTSD Symptom Scale; and functioning	NA

*Notes*: *DRC* Democratic Republic of the Congo, *UK* United Kingdom, *USA* United States of America, *NR* not reported.

## Results

Out of 5,684 returned records, we assessed 189 full text papers for eligibility (Figure 1). Most of these papers were excluded because they did not report any data, focused purely on assessment of sexual and gender-based violence, or described an intervention approach without mentioning an evaluation component.

Altogether, we identified 7 studies that met inclusion criteria (Table 1). One of these concerned a non-randomized controlled study; three applied non-controlled pre- post-test designs; one was a retrospective cohort design with a comparison group; and two were single case studies. More than half (n = 4) of these studies were conducted in West and Central Africa, two were conducted with refugees in the USA, and one was conducted in Albania. Studies included exclusively women and evaluated more generic multidisciplinary interventions (e.g. group counseling, support groups, combined psychosocial and economic interventions, medical care and psychological support), as well as a specialized psychotherapeutic intervention (i.e. cognitive behavioral therapy). The types of interventions studied confirm an earlier mapping of MHPSS interventions in humanitarian settings (including disasters) more broadly. This mapping found that individual and group counseling and structured social activities are among the most popular interventions for populations affected by humanitarian crises. Quality of studies ranged from 12 to 16 items out of 27, indicating significant limitations in study design and reporting.

Lekskes, van Hooren & De Beus compared the effectiveness of (a) individual and group counseling (n = 34 at follow-up) and (b) support groups and skills training (tie-dying fabrics, sewing and soap making; n = 22 at follow-up) with a waitlisted control group (n = 10 at follow-up). Sixty-eight percent of the participants reported exposure to sexual violence, with 19% reporting having been raped at least once, and 11% reporting gang rape. Both interventions were implemented in community settings by trained para-professionals employed by non-governmental organizations. Counseling consisted of at least 8 sessions of individual counseling and group counseling, focused on reducing stress and trauma with the group counseling covering themes of “stress management, conflict resolution, hygiene, and peace building” (p.21). Despite challenges in implementation, women reported being positive about the interventions in qualitative interviews. Quantitative results showed a decrease of PTSD symptoms in the counseling group, and slight increases in the support and waitlist conditions, but no statistical significance of these effects was reported. Although this study is the only controlled study of a popular counseling approach in armed conflict settings, the study has limitations including a lack of randomization, which is of particular concern given that women in the counseling condition reported higher exposure to sexual violence and levels of PTSD symptoms at baseline. In addition, authors report large loss of participants at follow-up (54.5%); implementation of the intervention in the context of other on-going socio-economic interventions in research communities; and a lack of specific training of counselors on issues of sexual violence and sexuality [36].

Bolton reports on a non-controlled mixed methods program evaluation of psychosocial activities implemented by the International Rescue Committee (IRC) in South Kivu, Eastern Democratic Republic of Congo (DRC). A qualitative study explored local concepts of psychosocial problems related to gender-based violence and functioning. Subsequently, 240 women participated in pre-program assessments, 200 of whom were assessed after the intervention, and 66 of whom participated in a follow-up measurement. At baseline, women reported particularly high levels of impairment in functioning (e.g. farming, trading, cooking, looking after children), anxiety and fear. The author reports that in addition to the lack of a control group, the type of interventions changed between the start and closure of the study -from a variety of economic and/or psychosocial interventions implemented by various partner organizations to a more structured intervention delivered by four partner organizations trained by the IRC, making it hard to infer which intervention was responsible for changes over time. Nonetheless, women reported substantial improvements in both functioning and symptomatology at follow-up assessments [37].

Hustache and colleagues report the evaluation of services implemented by Médecins Sans Frontières in conflict-affected Republic of the Congo, including medical services and psychological support. Psychological support, delivered by a psychologist, included offering a safe environment for sharing of experiences and expression of distress, active listening, normalizing reactions, work on coping strategies and development of future plans. Pre- and post-test comparisons of 59 women participating in at least 2 sessions (median 3) showed a decrease in ratings of severe impairment (from 22 participants with severe impairment pre-intervention, to 3 and 2 participants at end of treatment and 1 to 2 year follow-up respectively, p = .04) [38].

Plester describes the evaluation of group counseling for women in Albania implemented by a psychiatrist and social worker from Medica Tirana in a non-controlled pre-, post-test design. Counseling included a mix of psychodrama, cognitive behavioral therapy, imagination exercises and relaxation techniques over 10 to 12 sessions, and focused on talking about traumatic experiences or current difficulties experienced by women. The author reports significant decreases in PTSD symptoms and general psychological symptoms, and a non-significant increase in empowerment in a group of women persecuted and detained during the Communist regime, but no statistical tests are reported. Similarly to the study by Leskes, et al., participants with the highest PTSD scores improved the most [39].

Ager and colleagues report on a retrospective cohort design in Sierra Leone focused on a community reintegration program with girls and young female former combatants, implemented by Christian Children’s Fund. In the absence of pre-intervention assessments, girls and young women who received services (n = 74; randomly selected) were interviewed up to 5 years afterwards and were compared with girls in matched communities who did not participate in interventions (n = 68). Interventions included access to traditional cleansing ceremonies led by local spiritual healers; payment of medical services (e.g. for sexually transmitted infections); skills-training (soap making, tie-dyeing and crocheting); provision of micro-credits loans (e.g. to facilitate schooling and business activities); and raising awareness in communities on the plight of former female combatants. Measurements consisted of structured interviews focused on six local indicators of integration based on prior qualitative research. As many participants turned out to have achieved these indicators before implementation of the program, sub-analyses focused on matching intervention and comparison participants who had not achieved reintegration outcomes prior to program implementation. These analyses showed significantly more girls in the intervention condition achieving integration outcomes on five out of six indicators, including schooling, community acceptance, inclusion in traditional women’s initiation societies, cessation of drug use, and attainment of “steady head” (a locally developed indicator of mental stability), but not marriage [40].

Finally, both Vickers [41] and Schulz and colleagues [42] report on single case studies of cognitive behavioral therapy (CBT) for rape survivors with PTSD in the UK and USA. Vickers summarizes CBT with an unaccompanied minor from Africa (country not reported) who survived genocide, witnessed her mother’s killing, and survived rape. Therapy was implemented weekly in 16 sessions, with the client dropping out at the 17th session. Scores on the Post-Traumatic Diagnosis Scale went from 42 pre-treatment (severe PTSD) to 40 at session 6, 14 at session 15 and 9 (minor symptoms) at session 16. Treatment included cognitive restructuring and sleep hygiene; examining behavioral cycles; psychoeducation; imaginal exposure to trauma-related events and reviews [41].

Schulz and coworkers describe a phased approach to cognitive processing therapy (CPT) with a female 64-year old Bosnian refugee who was repeatedly sexually and physically assaulted by an acquaintance during the war in the former Yugoslavia, in addition to witnessing war atrocities, bereavement and losses during flight and resettlement. Assessments were conducted through clinical interviews and use of the PTSD Symptom Scale (PSS). Modification of the CPT protocol, applied in 25 sessions over 9 months rather than the standard 12 session protocol, included keeping a dream journal to record a repetitive nightmare during the exposure part of treatment, and the client continued taking an anti-depressant (paroxetine). Scores on the PSS went from 33 (severe PTSD) at pre-treatment, to 11 two months into treatment and 4 at post-treatment [42].

## Discussion

The aim of this systematic review was to summarize knowledge on the effectiveness of mental health and psychosocial support interventions for populations exposed to sexual and other forms of gender-based violence in the context of armed conflicts. Despite a systematic combing of the academic and grey literature, we identified only 7 studies addressing this important topic. None of the studies were RCTs. We did not attempt a meta-analysis of results because of the methodological limitations of the identified studies. Before discussing this body of literature, we note an important limitation of our study: we searched using English language keywords only and may have missed studies reported in other languages or those available through regional databases only.

With regard to the identified evaluation studies, an obvious conclusion is that the number of studies conducted does not match the significance of the problem. Despite reports of large amounts of people surviving sexual and other forms of gender-based violence in the context of armed conflicts [8], high levels of psychological distress and mental disorders in this population [9-11], and the increasing popularity of preventive and treatment strategies [7,13], very little is known about which intervention strategies are effective. Knowledge from other types of populations, for example from the data on disaster and conflict-affected populations more generally [14], may not be assumed to be generalizable because of the stigma and social and economic exclusion associated with surviving sexual violence. More specifically, we found no evaluation studies (a) with children below 14 years of age; (b) with male participants; (c) outside the limited geographical areas of West and Central Africa, Albania, and the US and UK; and (d) with survivors of intimate partner violence in conflict-affected areas, despite this being a more common form of gender-based violence than wartime rape.

In addition to their relative scarcity, it is difficult to draw any robust conclusions from the identified evaluation studies because of serious methodological limitations, as indicated by the scores on the quality assessments. Without randomization, it is possible that the larger decrease reported in the one study [36] that applied a control condition is a result of observed pre-treatment differences. Moreover, 6 out of 7 studies [37-42] did not apply a control condition, leaving open the possibility that any decrease in symptoms represents natural recovery or regression to the mean [43]. A recent study (published after the dates of this systematic review) concerning a randomized controlled trial of Cognitive Processing Therapy by Bass and colleagues, is the exception. This trial found that this form of psychotherapy, delivered in 1 individual and 11 group sessions, was associated with larger improvements in depression and anxiety, PTSD and function impairment compared to individual support [44]. In addition to showing the feasibility of rigorous randomized studies with this population, this study showed that treatment benefits were maintained at 6 months despite exposure to ongoing traumatic events.

In addition, the quantitative studies included in this review [36-39] suffered from a high loss to follow-up, introducing possible bias in favor of intervention if those for whom interventions were not beneficial dropped out. Furthermore, 4 out of 7 studies [37-40] evaluated combined interventions, e.g. medical and psychosocial interventions. Although such combined interventions address real-life needs, from a research standpoint it makes it difficult to disentangle the effectiveness of one intervention vs. another. Furthermore, it is hard to draw firm conclusions about efficacy and causation in fluid (post)conflict environments with research methodology and the participants themselves having to adjust in order to cope with the rapid socio-political and economic changes impacting upon them. The IRC program evaluation in the eastern DRC [37] describes how access issues related to security, staff turnover and program dropouts impacted significantly on the intervention outcomes and the evaluation methodology.

## Conclusions

Despite their limitations, the 7 studies together tentatively suggest potential beneficial effects of intervention. In addition, the studies suggest that evaluations can be conducted in challenging situations. This is further supported by the Bass et al. study published after this review was completed [44]. Counseling and strengthening community-based supports are among the most popular interventions in humanitarian settings [14], and the current evidence base – albeit very small – highlights that evaluation of these interventions can be feasibly conducted through partnerships with implementing organizations. Similarly, there is consensus on the importance of assessing the possibility to collaborate with traditional healers [13]. The study by Ager and colleagues [40] described here represents one of the few quantitative studies that actually investigates the effectiveness of traditional cleansing ceremonies on locally derived indicators of wellbeing. Such research is crucial to strengthen evidence and provide accountability to stakeholders in real-life settings.

More focused and intensive research efforts are necessary to isolate the effects of specific strategies to improve well-being and prevent or treat mental disorders in order to strengthen the knowledge base of effective practices for the regrettably large population of survivors of sexual and other forms of gender-based violence in the context of armed conflict. Given the mental health problems that many people suffer as a consequence of sexual and other forms of gender-based violence it is critical that more attention be paid to research and action in this area, both to improve the health and wellbeing of survivors but also to prevent further ill health and promote wellbeing.

## Competing interests

The authors declare that they have no competing interests.

## Authors’ contributions

WT VS MvO CGM conceptualized and designed the study. WT VS MCG CM conducted the searches and analyzed the data. WT wrote a first draft of the paper. VS MCG CM MvO CGM critically revised the manuscript, and all authors read and approved the final manuscript.
